# Radiation dose optimized lateral expansion of the field of view in synchrotron radiation X-ray tomographic microscopy

**DOI:** 10.1107/S0909049510019618

**Published:** 2010-07-09

**Authors:** David Haberthür, Christoph Hintermüller, Federica Marone, Johannes C. Schittny, Marco Stampanoni

**Affiliations:** a Institute of Anatomy, University of Bern, Switzerland; b Swiss Light Source, Paul Scherrer Institut, Villigen, Switzerland; c Institute of Biomedical Engineering, University and ETH Zürich, Switzerland

**Keywords:** X-ray imaging, computed tomography, synchrotron microtomography, SRXTM, field of view, radiation dose, lung, lung development, alveoli, acinus

## Abstract

Volumetric data at micrometer level resolution can be acquired within a few minutes using synchrotron-radiation-based tomographic microscopy. The field of view along the rotation axis of the sample can easily be increased by stacking several tomograms, allowing the investigation of long and thin objects at high resolution. On the contrary, an extension of the field of view in the perpendicular direction is non-trivial. This paper presents an acquisition protocol which increases the field of view of the tomographic dataset perpendicular to its rotation axis. The acquisition protocol can be tuned as a function of the reconstruction quality and scanning time. Since the scanning time is proportional to the radiation dose imparted to the sample, this method can be used to increase the field of view of tomographic microscopy instruments while optimizing the radiation dose for radiation-sensitive samples and keeping the quality of the tomographic dataset on the required level. This approach, dubbed wide-field synchrotron radiation tomographic microscopy, can increase the lateral field of view up to five times. The method has been successfully applied for the three-dimensional imaging of entire rat lung acini with a diameter of 4.1 mm at a voxel size of 1.48 µm.

## Abstract 2

Increasing the lateral field of view of tomography-based imaging methods greatly increases the acquisition time. This article presents scanning protocols to obtain high-resolution tomographic scans with large lateral field of view at greatly decreased acquisition time and thus reduced radiation dose while resulting in high-quality three-dimensional tomographic datasets.

## 1. Introduction

The functional respiratory lung unit, the so-called acinus, is defined as the complex of alveolated airways distal of a last purely conducting airway, the terminal bronchiole (Rodriguez *et al.*, 1987 ▶). The total of all acini forms the lung parenchyma, the area where the pulmonary gas-exchange takes place. While the structural development of the gas-exchange region including the alveolar septa is quite well characterized (Schittny & Burri, 2008 ▶; Schittny *et al.*, 2008 ▶; Mund *et al.*, 2008 ▶), the development of the three-dimensional structure of its functional unit, of the acini, has not been studied much owing to the lack of suitable methods.

It is our goal to study the branching pattern of the acinar airways as well as the airflow within it. Tomographic methods, in particular synchrotron-radiation-based tomographic microscopy, can access this kind of information non-destructively and non-invasively.

In order to visualize the thin sheets of tissue (alveolar septa) forming the gas-exchanging alveoli, a resolution of the order of 1 µm is required. An entire acinus is usually larger than the field of view of the tomographic microscope (Rodriguez *et al.*, 1987 ▶; Weibel, 2009 ▶), being the latest limited by the chosen optical configuration. Usually, a large field of view resulting in a large sample volume can only be acquired with low magnification and *vice versa*. Laboratory-based micro-computed tomography (µCT) stations could potentially be used to study acini, but the resolution of such systems is too low to resolve all alveolar septa. Even if µCT stations are catching up, synchrotron-radiation-based tomographic microscopy beamlines provide the necessary high resolution combined with unmatched image quality.

Up to now the price to pay for this high resolution was a limited field of view. For instance, at the TOMCAT beamline (Stampanoni *et al.*, 2007 ▶) at the Swiss Light Source, Paul Scherrer Institute, Villigen, Switzerland, the field of view at a 10× magnification (0.74 µm voxel size) is limited to 1.52 × 1.52 mm, insufficient for the imaging of entire acini at high resolution.

Increasing the field of view perpendicular to the rotation axis of the sample cannot easily be achieved by placing tomographic datasets next to each other. It is instead necessary to merge several projections overlapping the desired field of view prior to tomographic reconstruction. Obviously, to satisfy the sampling theorem, increasing the field of view also requires acquiring more projections, finally resulting in an increased acquisition time.

We developed such a method to merge several independently acquired sets of projections to increase the field of view of the resulting tomographic dataset. In addition, by optimization of the number of recorded projections, we established different scanning protocols with a user-defined balance between acquisition time and image quality.

Because the total acquisition time is directly linked to the radiation imparted to the sample, it is obvious that such protocols also affect radiation damage and constitute an important optimization tool for radiation-sensitive experiments.

## 2. Materials and methods

### 2.1. Sample preparation

Rat lung samples, prepared according to Tschanz & Burri (2002 ▶) and Luyet *et al.* (2002 ▶), were used as test objects. Briefly, lungs of Sprague-Dawley rats were filled with 2.5% glutaraldehyde [CH_2_(CH_2_CHO)_2_] in 0.03 *M* potassium phosphate buffer (pH 7.4) by instillation *via* tracheotomy at a constant pressure of 20 cm water column. In order to prevent recoiling of the lung, this pressure was maintained during glutaraldehyde fixation for a minimum of 2 h. Subsequently, the lungs were dissected free and immersed *in toto* in the same fixative at a temperature of 277 K for at least 24 h.

The samples were postfixed with 1% osmium tetroxide [OsO_4_] and stained with 4% uranyl nitrate [UO_2_(NO3)_2_] to increase the X-ray absorption contrast, dehydrated in a graded series of ethanol and embedded in paraffin using Histoclear (Merck KGaA, Darmstadt, Germany) as an intermedium. The lung samples were mounted onto standard scanning electron microscopy sample holders (PLANO GmbH, Wetzlar, Germany) using paraffin (Tsuda *et al.*, 2008 ▶).

The handling of animals before and during the experiments, as well as the experiments themselves, was approved and supervised by the Swiss Agency for the Environment, Forests and Landscape and the Veterinary Service of the Canton of Bern, Switzerland.

### 2.2. Synchrotron radiation tomographic microscopy

The experiments were performed at the TOMCAT beamline at the Swiss Light Source, Paul Scherrer Institut, Villigen, Switzerland. The samples were scanned at 12.6 keV. After penetration through the sample, the X-rays were converted into visible light by a YAG:Ce scintillator (18 µm thickness, Crismatec Saint-Gobain, Nemours, France). Projections were magnified by diffraction-limited microscope optics (10× magnification) and digitized by a high-resolution 2048 × 2048 pixel CCD camera (pco.2000, PCO AG, Kelheim, Germany) with 14-bit dynamic range. The detector was operated in 2 × 2 binning mode. As a result, the pixel size was 1.48 µm and the exposure time was 175 ms.

Projections *I*_Pr_ were recorded at equiangular positions between 0° and 180°. The exact number of angular projections depended on the selected scan protocol, as described in §2.3. Additionally, for each protocol a set of dark (*I*_D_) and flat images (*I*_F_) were recorded for noise and baseline correction, respectively. Technical specifications of the beamline set-up can be found by Stampanoni *et al.* (2006 ▶); the complete imaging and reconstruction workflow is described by Hintermüller *et al.* (2010 ▶).

### 2.3. Increasing the field of view

**Figure 1:**
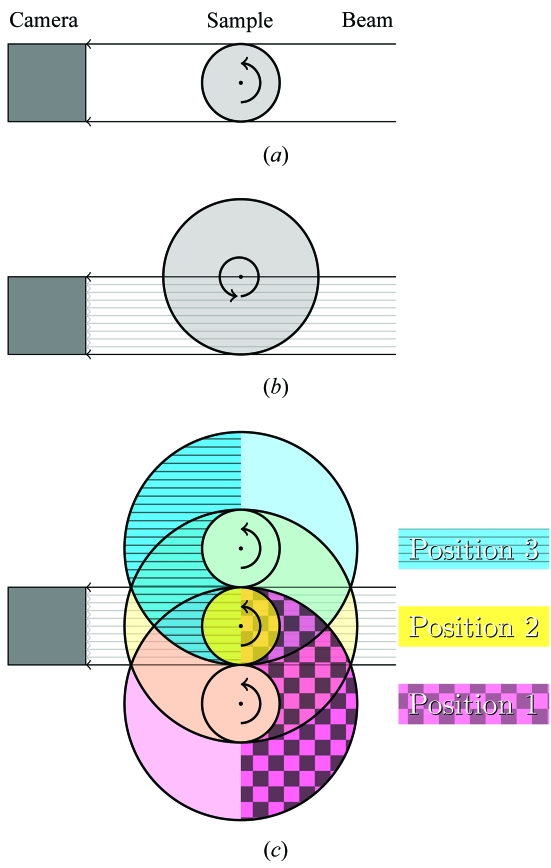
Covering the field of view of differently sized samples with one 180° scan (*a*), one 360° scan (*b*) or, in the case of the so-called wide-field scanning, with multiple subscans (three subscans, *c*). The filled segments mark the region of the sample that is covered while scanning the respective positions (position 1: magenta/checkerboard; position 2: yellow; position 3: cyan/striped).

For parallel-beam geometry, tomographic images are obtained at equidistant angles over a sample rotation of 180° as shown in Fig. 1(*a*) ▶. After reconstruction, the width of the image corresponds to the field of view of the camera.

Samples twice as large as the field of view can be imaged using scanning protocols based on a 360° off-center sample rotation as shown in Fig. 1(*b*) ▶. Images recorded between 180° and 360° have to be flipped after acquisition: the projections obtained at angular position θ and θ + 180° (*I*_Pr_θ__ and *I*_Pr_θ+180°__) have to be stitched to one projection. The resulting images cover twice the field of view of the camera.

For tomographic scans covering a size wider than two fields of view, three or more 180°-scans taken at slightly overlapping positions are combined, as shown in Fig. 1(*c*) ▶. The projections of each subscan overlap slightly to facilitate the stitching of multiple projections into a single one. The cutline, *i.e.* the position where the merging takes place, is automatically determined according to a mean-squared difference method (Hintermüller *et al.*, 2010 ▶).

A straightforward acquisition scheme would record an equal amount of projections for each of the individual subscans. As a consequence, to fulfill the sampling theorem in the lateral parts of the sample, oversampling the central parts of the sample would be necessary.

Since the total acquisition time per sample linearly scales with the total amount of recorded projections, such an acquisition scheme obviously increases the total amount of beam time for one sample without relevantly increasing the quality of the reconstructed tomographic data. Hence, such an oversampling is generally avoided.

**Figure 2:**
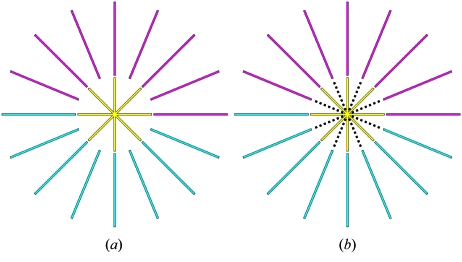
Wide-field scan set-up with three 180° scans; one central (yellow) and two lateral scans (magenta and cyan or top and bottom, respectively). In this drawing, four projections for the central and eight projections for each of the lateral scans have been recorded. The colors of the three positions correspond to the colors shown in Fig. 1(*c*) ▶. (*a*) Scanned projections; (*b*) scanned projections and additional interpolated projections (dotted) required to merge all projections.

Our goal was to find a good compromise between scanning time and image quality. We therefore devised an acquisition scheme for covering a wide field of view based on the assumption that a sufficient resolution and contrast can be achieved in the tomographic dataset, if the sampling theorem is individually fulfilled for each of the subscans. This results in a set of *i* subscans with *P*_*i*_ projections each. A simple example with *P*_2_ = 4 and *P*_1_ = *P*_3_ = 8 is shown in Fig. 2(*a*) ▶. Since each subscan *i* has a different number of projections *P*_*i*_, the stitching algorithm has to interpolate missing projections from adjacent projections [represented by the dotted lines in Fig. 2(*b*) ▶] to generate a complete set of merged projections for reconstruction.

As a by-product, such an optimization of the individual number of projections *P*_*i*_ for each subscan *i* decreases the total acquisition time for one sample and thus the imparted radiation dose.

**Figure 3:**
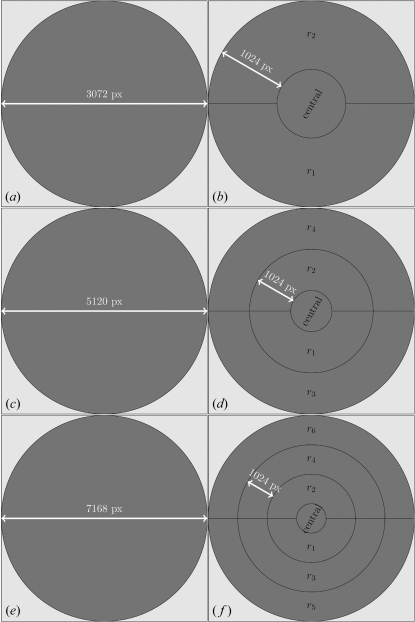
Set-up for different fields of view. (*a*) Desired field of view of 3072 pixel diameter. (*b*) Wide-field scanning protocol for covering the desired field of view of panel (*a*) with merged projections from one central and two half ring scans (*r*_1_ and *r*_2_). (*c*) Desired field of view of 5120 pixel diameter. (*d*) Wide-field scanning protocol for covering the desired field of view of panel (*c*) with merged projections from one central and four half ring scans (*r*_1_–*r*_4_). (*e*) Desired field of view of 7168 pixel diameter. (*f*) Wide-field scanning protocol for covering the desired field of view of panel (*e*) with merged projections from one central and six half ring scans (*r*_1_–*r*_6_).

We defined a gold standard protocol and several additional scanning protocols in order to compare different acquisition schemes. The gold standard protocol covers the desired field of view while fulfilling the sampling theorem, which states that for a detector width of *D* pixels we need to acquire a number of projections *P* = *D*π/2 (Kak & Slaney, 1988 ▶), in all its regions, as shown in Fig. 3(*a*) ▶. In this case we need to achieve a field of view of 3072 pixels. The dark gray circle is the field of view that could be covered using a large detector with a size of 3072 pixels and recording *P* = 3072π/2 = 4825 projections.

Using a detector with a size of 1024 pixels, this desired field of view could be covered with nine independent local tomography scans. Such an approach would require nine independent reconstructions and stitching of those nine reconstructed tomographic datasets into one dataset covering the full field of view. This method would also introduce artifacts at the edges of each of the nine sub-datasets which would lie inside the sample to be imaged.

While the chosen field of view of 3072 × 3072 pixels can be covered using a detector of size 3072 pixels in one scan, we can cover the desired field of view with a much smaller detector, using a scanning protocol with three subscans from which we obtain merged projections. Fig. 3(*b*) ▶ shows how the desired field of view of 3072 pixels can be covered with a wide-field scan, composed of one central and two half ring-scans, recorded with a small detector with a size of 1024 pixels and 4825 projections per subscan (a total of 14475 projections) which are then subsequently merged to 4825 large projections spanning the whole field of view. A further increase in the field of view can be obtained by simple iteration. Figs. 3(*c*)–3(*f*) ▶ show such a set-up for a five- or seven-fold increase.

### 2.4. Quality guided protocols

Taking into account the experimental constraints such as desired field of view, available detector size, magnification and binning, a *MATLAB* script calculates a set of acquisition protocols. Each such protocol contains the number of projections for each subscan linearly scaled in total amount of projections from a gold standard scan down to a protocol where the sampling theorem is far from being satisfied (Table 1 ▶). Through optimization of the number of recorded projections, a reduction of the total acquisition time by 84% (compared with the gold standard) was achieved.

Using a Shepp–Logan phantom (Shepp & Logan, 1974 ▶) with added Gaussian noise as a reference image, a simulated tomographic scan and subsequent reconstruction was calculated for each of these acquisition protocols. For each protocol we calculated the expected reconstruction quality using the difference image between the reconstruction of this protocol and the initial reference image. This simulated reconstruction quality was plotted against the total acquisition time (red dots in Fig. 6).

The end-user, balancing between acquisition time and desired image quality, chooses one protocol from the presented set for scanning his sample. A file containing all the details of the chosen scan is written to disk, and parsed using a custom Python-script. This script interacts with the hardware control system at the TOMCAT beamline enabling an automated unattended batch acquisition of all necessary subscans.

To assess the simulations in a real-world example, we selected 19 different acquisition protocols with varying number of projections to scan one single sample (details are specified in Table 1 ▶, including the calculated quality for each protocol).

A scan covering the chosen field of view with nine independent local tomography scans, each with a field of view of 1024 × 1024 pixels, would need a total of *P* = 9(1024π/2) = 14476 projections. This protocol was not considered for this study, since the sampling theorem can be equally satisfied by acquiring the required amount of projections with one central and two ring scans, as defined in §2.3. Including an overlap of 100 pixels between the central and the ring scan, an equivalent wide-field scanning protocol (Protocol *A* in Table 1 ▶) requires the acquisition of 13534 projections [*P*_*A*_ = 3(3072 − 200)π/2].

Protocols *B*–*T* have been linearly scaled down with a decreasing number of acquired projections of the ring scans. To simplify interpolation and merging of the projections from each subscan, we only selected acquisition schemes where the number of projections of the inner and the outer subscans is the same or a multiple of two (see Fig. 2 ▶). This constraint also led to a slight oversampling for protocol *B*, otherwise the number of projections for each subscan of this protocol (5244 = 6 × 874) would not have scaled down nicely to the 874 projections used for protocol *T*.

All parameters of each protocol and each subscan (sample-position in relation to the beam, rotation angles and number of projections) were set in a preference file, generated using the aforementioned *MATLAB* script. One rat lung sample was scanned using each of the 19 different protocols (*B*–*T*), without manual intervention, permitting a direct comparison of the reconstructed datasets.

### 2.5. Projection merging and tomographic reconstruction

After acquisition of the three subscans per protocol, custom *MATLAB* functions read the parameters of the single subscans (*e.g.* sample name, amount of subscans, amount of dark and flat images) as well as the desired output-name and -suffix, and performed all necessary calculations, including loading of the correct projections from each subscan; normalizing; interpolation; cutline detection; correct stitching of the images into wide-field projections; and writing these merged projections as well as log files needed for the reconstruction to disk.

The merged projections were subsequently rearranged into sinograms, where the *n*th sinogram is composed of the *n*th line of every corrected projection. The *n*th slice of the tomographic scan was reconstructed from the *n*th sinogram using an FFT-based regridding algorithm (Dowd *et al.*, 1999 ▶; Marone *et al.*, 2008 ▶). The 19 tomographic datasets were reconstructed on a computing cluster composed of five 64-bit Opteron machines with four cores and 8 Gbyte RAM each. The reconstructions resulted in an image stack covering a large sample volume of 2792 × 2792 × 1024 pixels, a ninefold increase from the standard volume of 1024 × 1024 × 1024 pixels for one conventional scan.

## 3. Results

### 3.1. Image merging and reconstruction

**Figure 4:**
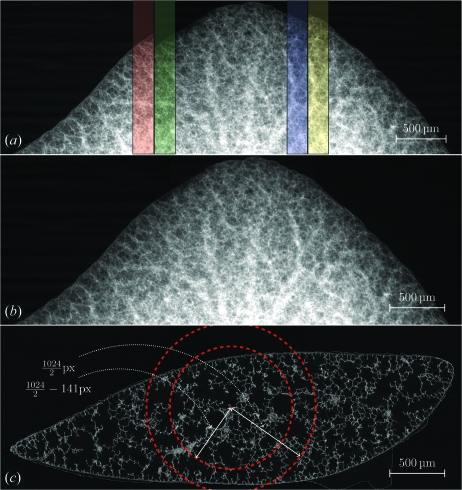
Workflow of a wide-field scan. The images show a rat lung sample from a Sprague-Dawley rat, obtained 21 days after birth, scanned with the acquisition protocol *B* (Table 1 ▶). (*a*) Three corrected and independently acquired projections from subscans *s*_1_–*s*_3_ are shown. Each one is 1024 × 1024 pixels large and covers a field of view of 1.52 mm. Subscans *s*_1_ and *s*_2_ overlap by 141 pixels (red and green overlay), subscans *s*_2_ and *s*_3_ overlap by 138 pixels (blue and yellow overlay). (*b*) Merged projection obtained from the three subscans shown in subfigure (*a*). Each merged projection has a size of 2792 × 1024 pixels. Owing to the overlap required to merge the projections, the width of the merged projections is slightly smaller than three times the width of the subscans. (*c*) Cropped slice of the reconstructed tomographic dataset. The dashed red circles mark the start and end of the overlap region.

Fig. 4(*a*) ▶ shows corrected projections from three overlapping subscans prior to merging, including regions where the subscans are overlapping. Fig. 4(*b*) ▶ shows one merged projection prior to reconstruction and Fig. 4(*c*) ▶ shows one slice of the reconstructed dataset. The example shown in Fig. 4 ▶ was obtained using the highest number of projections and is therefore protocol *B*. One reconstructed slice covers a field of view of 2792 × 2792 pixels (4.13 × 4.13 mm), which is almost three times the size of what can be achieved with one single-binned scan (1024 pixels or 1.52 mm). The dashed circles on the reconstructed slice mark the start and the end of the overlap region.

**Figure 5:**
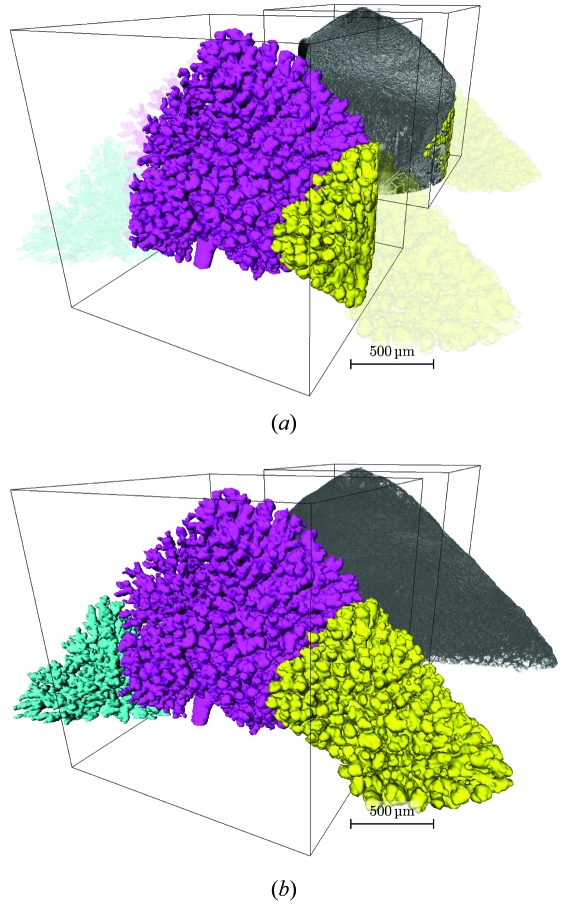
Three-dimensional visualization of the distal-medial tip of the right lower rat lung lobe. The gray structure in the background shows a semi-transparent view of the tomographic dataset with segmented airways. The foreground shows isosurfaces of terminal airways. The wireframe cube has a side length of 1024 pixels and encloses the field of view of one conventional scan. (*a*) Conventional scan; the extracted airway segments (magenta and yellow or left and right, respectively) are only partially contained inside the total sample volume. Airway segments not contained in the dataset but present in the sample are shown semi-transparent. This conventional scan corresponds to a reconstruction of the central of the three wide-field scan subscans. (*b*) Wide-field scan with increased field of view; the magenta (center) and yellow segment (right) show entire acini inside the dataset; the cyan segment (left) contains a partially cut acinus. All airway segments inside the sample are contained in the tomographic dataset.

Fig. 5 ▶ shows the advantages of the wide-field acquisition scheme. With, in this particular case, an enlargement of the field of view by almost a factor of three, it is possible to visualize entire acini at high resolution. For a conventional scan (Fig. 5*a* ▶), the airway segments in the sample are only partially contained inside the dataset (magenta and yellow). The semi-transparent airway segments are contained in the sample, but are not visible in the field of view of a dataset obtained with a conventional scan. Increasing the field of view (Fig. 5*b* ▶) allows the visualization of those segments to their full extent. A third acinus (cyan) which was not visible in Fig. 5(*a*) ▶ can now easily be visualized.

### 3.2. Performance of the scanned protocols

**Figure 6:**
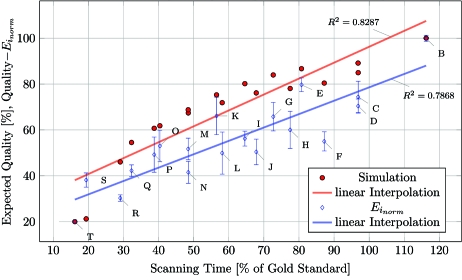
Plot of normalized difference value (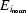, blue diamonds) for the 19 scanned protocols overlaid over quality-plot (red dots) obtained from the simulation (described in §2.4). The normalized error has been calculated using the difference image of each protocol *i* with protocol *B*. The error bars for each protocol show the standard deviation of the error calculated for 205 of the 1024 slices. Note that the scale of the error was normalized to 20–100%, so that both the quality from the simulation and the error are directly comparable. The abscissa shows the scanning time in percentage of time used for the gold standard scan. Protocol *T* on the far left corresponds to the fastest scanning time, protocol *B* on the far right to the slowest. The protocols in between are shown from *T*–*B* for increasing percentage of the scanning time.

The performance of the 19 protocols has been quantified using the difference image between binarized slices of the gold standard protocol and each protocol to be assessed. The slices have been thresholded according to Otsu (1979 ▶). The difference value (*E*_norm_) plotted in Fig. 6 ▶ was calculated for each protocol *i* = 1–19 (*B*–*T*) according to equations (1)–(3). Using a thresholded slice *k* of each protocol *i* (Slice_*i*_*k*__) and the corresponding slice *k* of the gold standard protocol *B* (Slice_*B*_*k*__), the absolute difference image (*D*_*i*_*k*__) of these two slices *k* was calculated. The sum of all pixels of this difference image yields a value (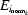) for the difference of the examined slice *k* of protocol *i* with the corresponding slice of the gold standard protocol *B*,
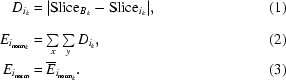
This combined difference value(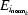) was calculated for 205 regularly spaced slices (every fifth slice) of the full dataset. The mean (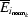) difference value for all slices was normalized to the scanned quality-steps from 16 to 116% (as stated in Table 1 ▶) and plotted with its standard deviation [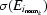]. For the purpose of comparison, data have been normalized.

As expected, the calculated quality of the reconstructions representing the different protocols decreases as a function of total number of obtained projections (Fig. 6 ▶). The calculated error of the different protocols (normalized difference value, blue diamonds) shows the experimental results obtained from actual scans of lung tissue. The plots for the simulation as defined in §2.4 (red dots) and the normalized difference value are not perfectly in agreement, but show the same trend. The linear regression for the simulation shows a steeper decrease for the quality (*y*_Sim_ = 0.6936*x* + 26.891) than the linear interpolation for the experimental data (*y*_Exp_ = 0.5833*x* + 20.226). The linear-regression coefficient for both the linear interpolations are comparable (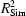 = 0.8287, 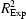 = 0.7868).

### 3.3. Three-dimensional visualization of different protocols

**Figure 7:**
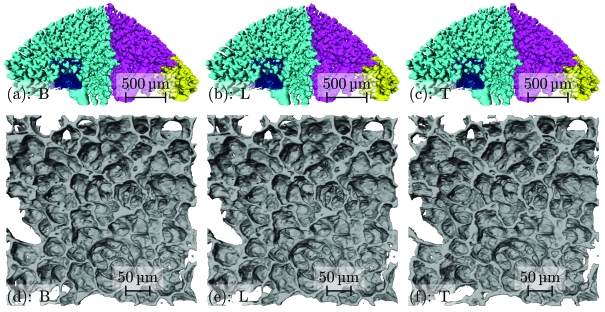
Comparison of three-dimensional visualizations. (*a*), (*b*), (*c*) Three independent airway segments (cyan, magenta, yellow) of tomographic datasets obtained with protocol *B*, *L* and *T*, extracted using a region-growing algorithm. A cubic ROI (blue) with a side length of 256 pixels (corresponding to 379 µm) is marked inside the leftmost segment for all protocols. (*d*), (*e*), (*f*) Detailed view of isosurfaces of the lung tissue inside the blue ROIs for protocol *B*, *L* and *T*, respectively. Note the increasing surface roughness in the alveolar surfaces for subfigures (*e*) and (*f*).

The tomograms of the different protocols were three-dimensionally analyzed and visualized using *MeVisLab* [Version 2.0 (2009-06-09 Release), MeVis Medical Solutions AG and Fraunhofer MEVIS, Institute for Medical Image Computing, Bremen, Germany]. Airway segments were extracted using a threshold interval-based region-growing algorithm (Zucker, 1976 ▶). A seed point for the region-growing algorithm was manually defined in the most proximal slice for each independent airway segment. The coordinates of the seed points were kept constant for protocol *B*–*T*, allowing direct comparison between the airway segment reconstructions of the different protocols. Airway segments extracted for protocol *B*, *L* and *T* are shown in Fig. 7 ▶.

Protocol *B* corresponds to a slightly oversampled gold standard scan, obtained with a total of 15732 projections, recorded in 66 min. Protocol *L* was obtained in 35 min with a total of 7866 projections. Protocol *T* was obtained in 12 min with 2185 projections for all three subscans. The tomographic dataset from protocol *B* was reconstructed from 5244 merged projections, the dataset from protocol *L* was reconstructed from 2622 merged projections, and the dataset from protocol *T* was reconstructed using only 874 merged projections. Even though protocols *L* and *T* were scanned while violating the sampling theorem and with a total scanning time reduction of 40% (*L*) or more than 86% (*T*), the samples still appear to be identical to the gold standard protocol in the low-resolution three-dimensional visualizations shown in Figs. 7(*a*)–7(*c*) ▶.

Figs. 7(*d*)–7(*f*) ▶ show isosurface visualizations of the border between airspace and lung tissue as cubic regions of interest (ROIs) [256 pixels wide, its location inside the sample is marked as a blue cube in Figs. 7(*a*)–7(*c*) ▶]. Because of experimental constraints, the cutline between the individual subscans could not be defined with a precision of one single pixel. As a consequence, the clipping plane does not lie in exactly the same position. This explains the appearing and disappearing holes in Figs. 7(*d*)–7(*f*) ▶.

Even with the higher magnification, the reconstruction of protocol *L* in Fig. 7(*e*) ▶ appears almost identical to the reconstruction of the ROI of protocol *B* (Fig. 7*d* ▶). The isosurface of the ROI of protocol *T* shown in Fig. 7(*f*) ▶ appears rougher than the isosurface of protocol *B*. This roughness is introduced through ray-like artifacts visible in the original slice of the dataset of protocol *T* (not shown). These artifacts are the consequence of a strong subsampling. With the acquisition of only 874 projections instead of the required 5139, the sampling theorem is far from being satisfied. However, even with this strong undersampling, segmentation, three-dimensional reconstruction and visualization of the sample is still possible.

**Figure 8:**
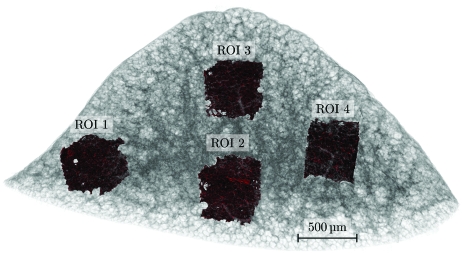
Overview of the location of the four ROIs where the histogram of the euclidean distance transformation distribution has been calculated. Gray: semi-transparent volume rendering of the lung tissue sample. Red: four ROIs, extracted to calculate the distance transformation. The labels of the ROIs conform to the legends in Fig. 9 ▶.

For further analysis, four ROIs with a side length of 256 pixels have been extracted for each of the protocols *B*, *L* and *T*. The three-dimensional location of these ROIs inside the sample is shown in Fig. 8 ▶.

Each of the ROIs has been binarized using an algorithmically determined threshold (Otsu, 1979 ▶) and small particles inside the segmented airspace lumen have been removed using a connected component analysis. Subsequently, the euclidean distance transformation (Danielsson, 1980 ▶) has been calculated for each thresholded ROI.

For comparison, the histogram of the euclidean distance transformation has been plotted for all four ROIs in each protocol (*B*, *L* and *T*).

**Figure 9:**
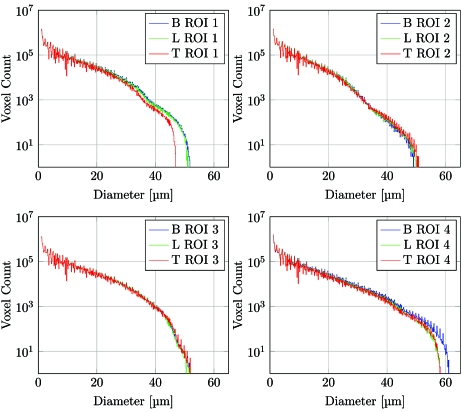
Histogram plots for each of the four ROIs, each showing the histogram of the distance transformation for the protocols *B*, *L* and *T*.

Fig. 9 ▶ shows logarithmic plots of the histogram distributions for the four selected ROIs; the blue, green and red plots show the histograms of the distance transformation of protocol *B*, *L* and *T*, respectively. For all four ROIs the distribution of the euclidean distance transformation is very similar; only for larger airway diameters (between 50–60 µm) do we see a detectable difference in the ROIs 1 and 4, located in the lateral parts of the sample. If we remember that the histogram is plotted with a logarithmic *y*-axis, we see that the difference in the histograms is only visible for several hundred voxels.

Even when reducing the sample acquisition time by 84% of the gold standard scan (*T* *versus* *B*), the distance transformation histograms of the shown ROI are very similar and therefore no relevant structural differences are introduced.

As a further proof of concept we scanned and reconstructed a rat lung sample with five scanning positions, resulting in an almost fivefold (4.74×) increase in field of view from slices with a size of 1024 × 1024 pixels to a size of 4852 × 4852 pixels (1.52 × 1.52 mm to 7.18 × 7.18 mm) at a voxel side length of 1.48 µm. A three-dimensional visualization of the boundary between airspace and tissue in this reconstructed dataset validated the wide-field scanning method for further increases in the available field of view (data not shown).

## 4. Discussion

We present a method to laterally increase the field of view of tomographic imaging systems operated in parallel-beam geometry and would like to call this method wide-field synchrotron-radiation-based X-ray tomographic microscopy (WF-SRXTM). We defined scanning protocols for the optimization of the total imaging time *versus* the expected imaging quality, enabling a very fast acquisition of lower quality tomographic datasets, or acquisition of very high quality datasets in a longer time.

Even if the reduction in scanning time does introduce minor artifacts in the three-dimensional reconstruction, as shown in Fig. 7 ▶, an automated segmentation of the relevant features in the sample is still possible, even for protocols with greatly reduced scanning time.

The introduced artifacts in the three-dimensional reconstruction of the lung tissue are of small scale compared with the alveoli, the smallest structures we would like to visualize. At our scale, the structures which are in the range of our resolution are holes visible in the alveolar septa. Those holes may appear through the three-dimensional reconstruction at locations where the alveolar septa are too thin and/or the globally chosen threshold is too high. However, the observed holes are not exclusively artificial: Kohn (1893) ▶ described micrometer-sized pores, so-called pores of Kohn, located between adjacent alveoli, which can also be seen in rat lungs (Van Meir, 1991 ▶).

Comparing the reconstructions shown in Figs. 7(*d*)–7(*f*) ▶ we observe a change in size of these pores. The pore size is affected by both the introduced artifacts and the algorithmically chosen threshold in these reconstructions.

Biologically interesting phenomena like emphysematic lung diseases introduce much larger defects in the lung tissue, where the size of the acinus is enlarged and the peripheral airways are collapsed (Weibel, 2009 ▶). Defects like these would still easily be detectable with an undersampled scan since the introduced artefacts are orders of magnitude smaller than the tissue alterations to be detected.

If other samples are to be observed using the proposed WF-SRXTM method, the desired level of image quality and therefore the corresponding reduction of the scanning time has to be defined according to the smallest structure present in the sample to detect.

The shorter scanning time obviously introduces minor artifacts in the reconstructed images but it is sometimes desirable, especially when radiation-sensitive samples need to be investigated. With a suitable protocol the dose can be reduced by 84% (Table 1 ▶), which might be a significant step towards tomographic imaging of sensitive samples using ultrahigh resolution and enhanced field of view.

The field of view was increased threefold by merging projections from three partially overlapping scans and reconstructing these resulting projections using the standard workflow at the TOMCAT beamline (Fig. 4 ▶). The high precision of the linear motors used to move the sample stage [resolution better than 1 µm in all three space directions, 0.1 µm accuracy perpendicular to the beam direction (Stampanoni *et al.*, 2006 ▶)] permitted a highly reproducible positioning of the lung sample for the consecutive scans.

The sample rotation stage of TOMCAT has a run-out error of less than 1 µm at 100 mm from the rotation surface (Stampanoni *et al.*, 2006 ▶). This precise angular positioning made it possible to merge the projections from the consecutive subscans recorded at the same angular step but differing lateral position into one projection spanning the large field of view. As a consequence of the sampling theorem, an increased amount of projections had to be acquired for an increase in the field of view, thus increasing the acquisition time. To overcome this limitation, we defined multiple scanning protocols with a reduced amount of total projections and thus reduced acquisition time and delivered dose (Table 1 ▶). All of these protocols were evaluated for the quality of the resulting reconstructions and compared with a gold standard scan. We have shown that the resulting quality can be simulated prior to scanning and thus provides a tool to choose a suited scanning protocol, based on the demands for scanning time optimization and quality of the resulting tomographic dataset (Fig. 6 ▶).

Reducing the amount of projections for the central of the three subscans may be performed with a minor loss of fidelity in the resulting reconstructions. Let us compare protocols *D*/*E* and *H*/*I*. For protocols *E* and *I* we acquired half the amount of projections for the central subscan *s*_2_ as compared with protocols *D* and *H*. In both cases we reduce the scanning time by 17%, but keep the quality of the scan on a comparable level (*D*: 70% ± 3.09 *versus* *E*: 80% ± 3.01, *H*: 60% ± 8.08 *versus* *I*: 56% ± 3.23). We show that the interpolation of missing projections does not introduce relevant errors in the resulting tomographic datasets.

For protocols with an equal amount of total projections, but differing amount of projections for the individual subscans (*C*/*D* and *M*/*N*), we observed minor differences in reconstruction quality. The qualities 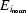 of protocols *C* and *D* lie within their respective standard deviation (74% ± 6.81 *versus* 70% ± 3.09), and the qualities of protocols *M* and *N* are comparable (52% ± 4.71 *versus* 42% ± 4.78). Both protocols *C* and *M* are scanned without oversampling the central subscan, making interpolation necessary; for protocols *D* and *N* we simply stitched the projections of the three subscans. Note that for protocol *N* we do undersample the outer parts of the sample. When deciding between two protocols with the same amount of total projections, it is thus desirable to favor the protocol where the central scan is not oversampled (*i.e.* choosing protocol *C* instead of *D*). Even if this introduces additional computing time to interpolate projections prior to reconstruction, these protocols show an increased quality compared with protocols where the central scan is oversampled. Since an oversampling of the central scan does not add much to the total reconstruction quality and the outer parts of the sample contribute more to the total area of the projections, choosing a protocol where the sampling theorem is satisfied better for those parts of the sample is favorable (*i.e.* favoring protocol *M* to protocol *N*).

With the defined protocols we open the possibility for the end-user to choose an acquisition mode suited to fulfill the constraints on number of samples to be scanned within the allocated beam time and desired quality of the reconstructed datasets.

Additionally, two special use cases for different protocols are worth mentioning. First, if the user needs a very quick overview over samples at high resolution, a time-saving protocol can be used. This is especially the case if the integrity of the sample can only be judged with a tomographic scan. Based on the quick scan the correct samples for high-resolution scans may be selected. It has to be mentioned that a quick overview could, in principle, be obtained with a low-resolution scan, which usually automatically accommodates a larger field of view. However, the resolution of such an overview scan is not always sufficient to detect interesting features in the samples which might be damaged.

We have shown that the field of view of parallel-beam tomographic end-stations can be increased up to fivefold and have routinely reconstructed multiple tomograms with a threefold increase in field of view. The shown acquisition protocols are theoretically expandable for more than five subscans, although the reconstruction of wide-field scans with seven or more subscans would require an extremely powerful data processing infrastructure. The datasets shown in Fig. 7 ▶ are binned scans resulting in datasets of 1024 slices, each with a size of 2792 × 2792 pixels at 8-bit gray value depth, which adds up to a total size of the dataset of approximately 7.5 Gbyte. If we assume an unbinned scan with seven overlapping subscans, the size of one stitched projection will be approximately 14000 × 14000 pixels. The full dataset will consist of 2048 such slices, which would add up to a total size for the full dataset of approximately 383 Gbyte.

Even if the amount of data to handle is huge, a wide-field scan with a fivefold increase in field of view remains interesting, since it would enable the end-user to selectively reconstruct only ROIs from large samples with ultrahigh resolution. Up to now, a two-step process was required to scan precisely defined regions from samples larger than the field of view. This process involved the use of different magnifications, two separate beam times and a precise registration of the samples between those beam times.

## 5. Summary

A method to increase the lateral field of view of tomographic imaging has been established, which enables the high-resolution tomographic imaging of large samples that are wider than the field of view of the optical set-up in multiple semi-automatically combined steps. Tomographic datasets of entire rat lung acini have been acquired with an enhanced field of view using WF-SRXTM.

Different optimized scanning protocols for covering a large field of view have been validated and are now provided for the end-users of the TOMCAT beamline. End-users now have the possibility to choose suitable scanning protocols depending on a balance between acquisition time and expected reconstruction quality. Depending on this balance, a reduction of the image acquisition time by 84% is possible, while keeping the quality of the reconstructed tomographic dataset on a level still permitting automated segmentation of the lung structure and surrounding airspace, as shown in §3.3. The reduction in acquisition time obviously reduces the time during which the sample is irradiated by synchrotron radiation and thus reduces the radiation dose inflicted on the sample.

## Figures and Tables

**Table 1:** Details of the 19 scanned protocols for this study (*B*–*T*) An unoptimized scan to cover the desired field of view of 3072 pixels with nine independent scans (with a detector width of 1024 pixels) would require recording a total of 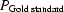 = 9(1024)π/2 = 14476 projections. The wide-field scanning protocol (*A*) equivalent to this field of view only uses three subscans, resulting in a total number of projections of *P*_*A*_ = 3(3072 − 200)π/2 = 13534. Three-dimensional reconstructions of the datasets marked in bold are shown in Fig. 7 ▶.

	Projections for subscan			Total number	Time/radiation	Simulated
Protocol	*s*_1_	*s*_2_	*s*_3_	of projections	dose (%)	quality (%)
*A*†				13534	100	
***B***‡	**5244 **	**5244 **	**5244 **	**15732**	**116 **	**100**
*C*	5244	2622	5244	13110	97	89
*D*	4370	4370	4370	13110	97	85
*E*	4370	2185	4370	10925	81	87
*F*	3934	3934	3934	11802	87	80
*G*	3934	1967	3934	9835	73	84
*H*	3496	3496	3496	10488	77	78
*I*	3496	1748	3496	8740	65	80
*J*	3060	3060	3060	9180	68	76
*K*	3060	1530	3060	7650	57	75
***L***	**2622 **	**2622 **	**2622 **	**7866 **	**58 **	**72**
*M*	2622	1311	2622	6555	48	69
*N*	2186	2186	2186	6558	48	67
*O*	2185	1093	2185	5463	40	62
*P*	1748	1748	1748	5244	39	61
*Q*	1748	874	1748	4370	32	55
*R*	1312	1312	1312	3936	29	46
*S*	874	874	874	2622	19	21
***T***	**874 **	**437 **	**874 **	**2185 **	**16 **	**20**

† Gold standard for this study.

‡ Wide-field scan equivalent to an unoptimized scan covering the field of view with nine independent scans.
